# Mortality of Beef Cattle During Long-Distance Transport by Ship or Truck

**DOI:** 10.3390/ani16050738

**Published:** 2026-02-27

**Authors:** Grisel Navarro, Erika Pavez-Muñoz, Clive J. C. Phillips

**Affiliations:** 1 Departamento de Medicina Veterinaria, Facultad de Recursos Naturales, Universidad Católica de Temuco (UCT), Temuco 4780000, Chile; 2 Escuela de Graduados, Facultad de Ciencias Veterinarias, Universidad Austral de Chile, Valdivia 5090000, Chile; erika.pavez@alumnos.uach.cl; 3 Programa de Bienestar Animal, Facultad de Ciencias Veterinarias, Universidad Austral de Chile, Valdivia 5090000, Chile; 4 Curtin University Sustainability Policy (CUSP) Institute, Curtin University, Perth, WA 6845, Australia; clive.phillips58@outlook.com; 5 Institute of Veterinary Medicine and Animal Sciences, Estonian University of Life Sciences, 51014 Tartu, Estonia

**Keywords:** animal welfare, calves, heifers, cull cows, road, maritime

## Abstract

Patagonia in the far south of Chile is used as a breeding ground for cattle, which are then transported north for their final stages of growth before being killed for meat. The objective of this study was to compare mortality in beef cattle exposed to two different types of long-distance transport: by ship, using a roll-on/roll-off system, or by truck. Data from a major transport company were used to compare the mortality risks for calves and older cattle when conveyed by sea or road from southern to central Chile journeys of four and two days, respectively. Mortality was higher for both calves and older cattle traveling by sea than those traveling by road, but when the data was corrected for journey duration, mortality/day was greater for those traveling by road. Mortality was increased at high stocking densities and, for older cattle, winter was a high-risk season, compared with summer. Mortality was also higher for calves than older cattle. The results identify the greatest risks of mortality over the two routes to facilitate reform of the transport processes so that cattle welfare is improved.

## Simple Summary

Long-distance transport is an essential but challenging component of beef cattle production in Chile, because animals are regularly moved about 1500 km north from Patagonia in the far south for fattening or slaughter. During the transport, cattle may be exposed to multiple stressors, including fatigue, floor movement, poor environmental conditions, and prolonged absence of feed and water, which can compromise animal welfare and, in some cases, lead to mortality. Using records from a commercial transport company, we compared mortality in beef cattle during long-distance transport by ship (a 3–5 day roll-on/roll-off system) or by truck (a continuous 2 day journey). Mortality risk was influenced by transport method, animal type, season, and stocking density. In total, more deaths occurred when the cattle were transported by sea, but daily mortality rates were higher during road transport. Calves showed a higher mortality risk than older cattle, as did those transported in winter and at high stocking densities. These findings identify the main risk factors in this long-distance transport and provide evidence to inform improvements in transport management and regulatory frameworks aimed at reducing mortality and enhancing animal welfare in Chile.

## 1. Introduction

The transportation of livestock is a critical component of the global cattle industry [1,2,3]. Long-distance transport is common in countries like Chile, where breeding grounds are remote from feedlots systems or processing facilities. In Chile, many cattle are reared in Patagonia on a pasture of low nutritive value [4,5]. The severe weather in this region favors extensive grazing, which results in lean carcasses with poor muscularity [6]. Consequently, live animals are transported from Patagonia to the more temperate central-southern zone of Chile for fattening and/or slaughter [4]. Although transportation is essential for maintaining supply chains and a viable cattle industry, it is also one of the most stressful events in the production cycle [1,7]. The stressors include physical fatigue, thermal stress, a novel and often polluted environment, constant but often unpredictable floor motion, high stocking density and close proximity of unknown herd mates, and disruption of feed and water intake [8,9,10].

Among the risks encountered during the journey, mortality is the most severe outcome, which has both ethical and economic implications [1,7]. Research in many countries has highlighted the factors that contribute to an increased risk of death during long journeys [11]. These include transport duration, vehicle type, stocking density, environmental conditions, and the animals’ age and health [11,12]. Most studies have been conducted in Europe and North America, confirming increased mortality rates during long journeys [12,13,14]. During long-haul transport, calves and cull cows are more likely to die or at least become non ambulatory, compared with steers that are able to cope with the stress better [12].

The unique geographical characteristics of Chile, with some remote agricultural regions like Patagonia in the far south, present distinct challenges for livestock transportation. The transportation of calves and sheep over thousands of kilometers presents several possible routes, including both maritime and terrestrial transport [15,16], which allows for a comparison of sea and land transport. Both have significant hazards, the rough seas of the Golfo de Penas for the maritime route, and two crossings of the Cordillera de los Andes, and a portion of the journey through Argentina, for the terrestrial route. Despite the necessity of long-distance transportation in Chile, there is little research addressing the factors influencing mortality during these journeys.

Therefore, the objective of this study was to evaluate the risk factors associated with the mortality of cattle during long-distance transport from Patagonia to the Los Lagos region under commercial conditions, with particular emphasis on animal type, transport method, climatic conditions, and stocking density. Two alternative transport methods were compared: by sea, with short truck journeys to and from the boat, and a road-only route. This study aimed to quantify the relative contribution of these factors to mortality risk and to provide evidence to support improved management practices and policy decisions focused on reducing transport-related mortality, improving animal welfare, and enhancing the sustainability of the cattle industry.

## 2. Material and Methods

The study utilized retrospective data from 2020 to 2024, inclusive, that was provided by a transportation company that commercially transports cattle in the south region of Chile. The identity of the transportation company is confidential; however, it has more than twenty years of experience in livestock transport, and written informed consent was obtained, authorizing the use and publication of the data (Appendix A). Ethical approval for the study was obtained from Universidad Católica de Temuco, approval number CEIUCT0515001/23. There were 395 commercial voyages in total, 178 by sea (roll-on/roll-off ferry) and land (treatment sea) and 217 entirely by land (treatment land). Over the five-year study period, 22,429 animals were transported, classified as calves (i.e., recently weaned cattle, less than 8 months old), steers and heifers of 8–12 months of age, and older animals (steers, heifers and cows, more than 12 months of age). A summary of the specifications of the trucks (single and double deck) used for transport and the number of animals/truck is presented in Table 1. The trucks consisted of single wagon units, with side ventilation ports, three axles, and either single or double deck configurations. Only two were covered and the remaining 11 were uncovered.

Sea transport lasted 3–5 days, depending on weather conditions, covering approximately 1550 km between Puerto Natales in Chilean Patagonia and Puerto Montt, with a mean journey duration of 96 h. Cattle were collected from farms in Patagonia and traveled by truck to Puerto Natales for a mean distance of 100 km and a duration of 2 h. Sea transport used a roll-on/roll-off system, in which the trucks carrying the livestock were loaded onto and secured within a ferry for all trips, a seven deck passenger/cargo ferry built in 2022 with a cruising speed of 13 knots (23 km/h). The animals remained onboard the vehicles on the lowest deck throughout the voyage. During transport, animals received limited feed (one 20 kg bale of grass hay per truck twice daily) and restricted water, supplied either in half-barrel containers (uncovered trucks) [17] or via a hose directed at the animals’ mouths (covered trucks) twice daily. On arrival at Puerto Montt, trucks traveled approximately 110 km for a duration of 1.5 h.

**Figure 1:**
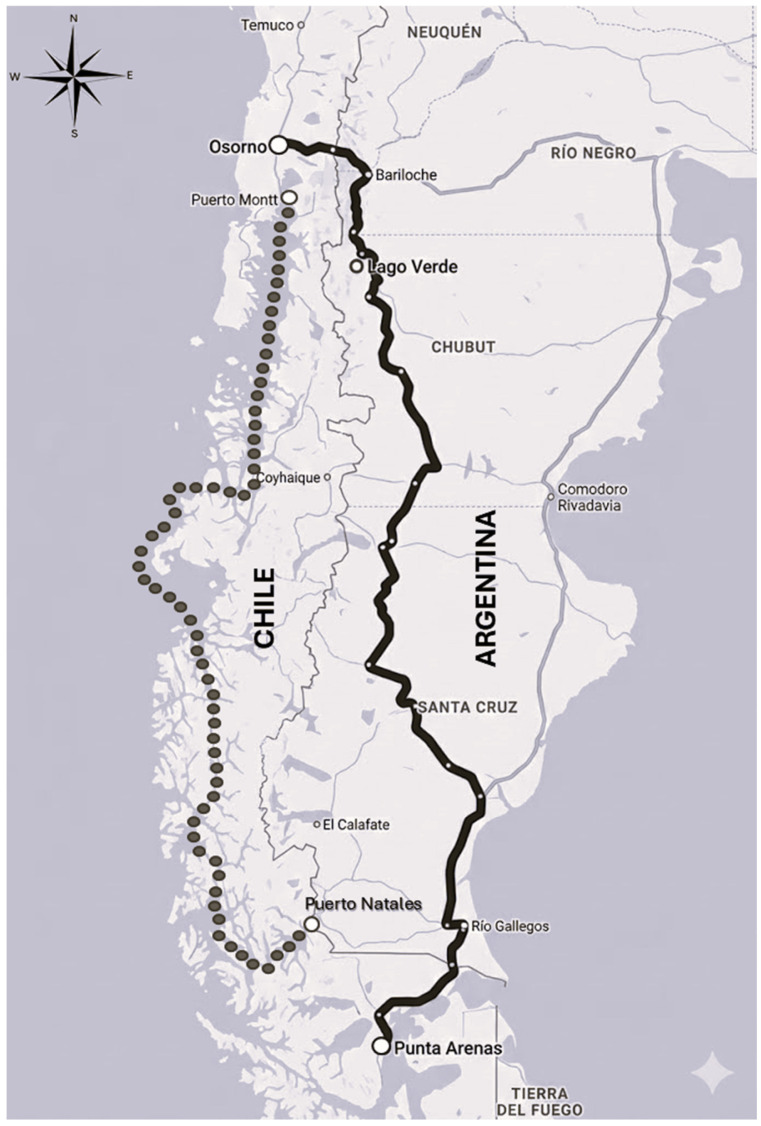
Map showing the two long-distance cattle transport routes evaluated in the study. The dotted line denotes the sea route (……) from Puerto Natales to Puerto Montt, and the land route (_______) departing from Punta Arenas, crossing Argentina, and re-entering Chile near Osorno.

The road trip lasted 2 days on average (1882 km, 57 h mean, average speed 33 km/h), starting in Punta Arenas, crossing to Santa Cruz, Argentina, through the international border crossing Paso Integración Austral, crossing back to Chile through the international border crossing Complejo Cardenal Samoré, and finishing in farms surrounding Osorno (Figure 1). For biosecurity reasons, the cattle could not be provided with feed or water during their road transit through Argentina, since their compartments were sealed by the relevant Argentinian sanitary authority on crossing the border. During the period of this study, no hay or straw originating in Chile was allowed to enter Argentina.

Mortality was recorded at the end of each journey, during unloading at the destination. The data were initially documented by the transporter on paper forms and subsequently digitized and entered into an Excel database. In the event of a death, the animal’s individual identification number (DIIO) was recorded, and the corresponding departure weight was adjusted by subtracting it from the total transported weight.

Mean seasonal climatic conditions in the transport regions were: summer 11–15 °C and 61–77% relative humidity (RH); autumn 5–10 °C and 73–84% RH; winter 4–8 °C and 72–82% RH; and spring 9–11 °C and 61–77% RH (Appendix A Table A1. Source: Agrometeorological Network of Instituto de Investigaciones Agropecuarias (INIA), Chile).

## 3. Statistical Analysis

We constructed a database with records of all performed trips, including the type of travel (by sea or over land), the type of truck, the number of animals that were transported and their live weight, the category of the animals (juvenile or adult), the sex of the animals, and the number of dead animals at the end of the journey.

An initial analysis ascertained the probability of death during long-distance transport. Given the low number of trips resulting in animal deaths, the initial approach was to categorize this variable dichotomously, as trips that did or did not result in animal deaths during execution. Logistic regression models were tested, but this revealed the presence of multicollinearity, and it was determined that the data had to be separated into two categories of animals. Two separate models were created for trips made with calves and older animals (i.e., steers, heifers and cows).

The models considered the mode of transport (sea or land), the type of truck (single deck or two decks), the season of the year (summer, autumn, winter or spring), the number of animals transported (n), the availability of space per animal (m^2^/animal), the total weight of animals transported (kg) and the stocking density in kg/m^2^ as factors. We developed initial models incorporating all the fixed factors, and subsequently removed them systematically from each model by a stepwise backward elimination process, until it had no significant effect on the response, or when, after removal, they did not modify the effect of the other variables by more than 15%. The final model was the one with the lowest Akaike Information Criterion value. Subsequently, the Hosmer–Lemeshow test was used to assess the goodness-of-fit of the model, in addition to evaluating the model performance through the ROC curve and AUC.

A second analysis was performed to determine the difference in the number of deaths corrected by the duration of the trip. The analysis was performed by correcting the 65 trips that resulted in one or more dead animals, according to the average duration of each trip category. In this model, land trips were assumed to take two days and sea trips four days. As this data was not normally distributed, a Wilcoxon Rank test was performed. All analyses were performed in Rstudio, using the latest available version (Ver. 4.4.2. R Foundation for Statistical Computing, Vienna, Austria, https://www.R-project.org/ (accessed on 15 November 2022)).

## 4. Results

None of the 395 trips had to be removed from the study for data quality issues, and of the 395, 65 resulted in at least one fatality. A total of 89 deaths were recorded, giving an overall mortality rate of 0.396%. Thirty were associated with road transport (0.270%) and 59 with maritime transport (0.519%). Of the 178 sea and 217 land journeys, sea trips occurred mainly in autumn (71.4%), with fewer in winter (18.5%), spring (6.7%), and summer (3.4%). land-based trips also mostly occurred in autumn, but not to the extent of sea trips (47.5%), with the next most common also being winter (20.3%), and more in summer (18.4%) and spring (13.8%) than sea trips. There were 259 trips with calves [144 by sea (10,188 calves) and 115 by land (7074 calves)] transporting a total of 17,262 calves. For older cattle, there were 136 trips [34 by sea (transporting 1160 cattle, 679 steers and heifers in 16 trips + 481 adult cattle in 18 trips) and 102 by Land (transporting 4007 cattle, 2278 steers and heifers in 48 trips + 1729 adult cattle in 54 trips)], making a total of 5167 steers, heifers, and adult cattle (Table 2).

The final model for calf journeys included the type of trip and the space availability per animal. The probability of death was less for calves in land transportation than for those transported by sea (odds ratio (OR) = 0.267, 95% confidence interval (CI) = 0.123–0.538, *p* < 0.001). Thus, the likelihood of death for calves during land transportation was approximately 73% lower than for those transported by sea (Table 3). Space availability was not statistically significant (OR = 0.307, 95% CI = 0.041–2.175, *p* = 0.198; Table 2), but was maintained in the model for a better fit.

In 39 out of 144 calves’ sea trips there was at least one mortality (27.08%); and in 11 out of 115 by land (9.57%). Out of 65 trips with a mortality, calves represent 76.92% (50 trips with a mortality). The final model for the older cattle incorporated the mode of transport, season, and stocking density (kg/m^2^; Table 4). Land trips were again associated with a significantly lower probability of death compared to sea trips, indicating a marked reduction of 89.1% in the likelihood of death during terrestrial transportation. There was a significant effect of the season in which the transport occurred, with winter exhibiting a higher probability of death in comparison to summer (OR = 23.11, 95% CI = 2.742–535.612, *p* = 0.012). The weight of cattle transported per unit of space was positively associated with the probability of death [18]; thus, increasing the weight transported per square meter elevated the risk of mortality.

In 9 out of 34 sea trips, there was at least one mortality (26.47%), and in 6 out of 102 by land (5.88%). Out of the 65 trips with mortality registered, trips transporting juvenile and adult cattle represent the 23.07% of the total trips with mortality (15 trips).

### Mortality Values Corrected for the Duration of Each Type of Trip

While sea transport recorded a higher absolute number of deaths, the time-adjusted analysis revealed the opposite pattern: daily mortality was substantially higher in Road transport, with corrected averages being approximately three times greater (0.88 daily deaths/trip) than those observed during sea journeys (Table 5). The calves had the highest corrected daily mortality rates, especially in Road transport (*p* < 0.001). In contrast, sea transport exhibited lower corrected daily mortality rates across both age categories.

## 5. Discussion

Millions of farm animals are transported every year around the world, most to be killed for meat but also for breeding [18,19]. The number of animals that die during the transport or shortly after the end of the journey is a critical indicator of animal welfare during this process [10]. The overall mortality rate, 0.4%, is greater than that reported for ship journeys exporting cattle from Australia, which was 0.07% for the period of this study [19]. The mean duration of ship transport from Australia is longer than in our study, typically one week, with a range of 4–30 d, and journey duration is recognized as being a risk factor for the welfare of heavy cattle, with greater space allowances required by the Australian Government on voyages of ten days or more [20].

The mortality rate of cattle in our study (0.4%) is higher than the mortality rate recorded for a relatively small sample of calves being transported to and from markets in Chile, which was 0.076%, with the transport being for only one or two hours on average and often on unpaved roads [15]. The difference is likely to be due to the fact that only calves were transported in the market study [15]. Our cattle mortality was higher than for cattle arriving at export abattoirs after truck transport in Australia in 2020/2021 (0.133% [21]). The latter study had a median duration of transport of only 5 h, compared with the mean of 2–4 days in our study. The Australian study [21] found that, as well as cattle being dead on arrival, there were many more cattle arriving with welfare issues: 33 times as many. The most common issues were handling problems, lameness, other injuries and blindness. Thus, the scale of welfare problems is not fully evident from mortality data. The reason for the comparatively high rate of mortality during the long-distance trips from Patagonia to central Chile is not known, as no other welfare data was collected; however, it is likely to relate to the limited supply of feed and water during the journeys.

In our study, calves were more likely to die during the long-distance transport than the older cattle (i.e., steers, heifers and cows; Table 4). This agrees with Gonzalez et al. [12], who indicated that calves and cull cattle are at a higher risk of mortality and of becoming non-ambulatory during transport. It is also consistent with Simova et al. [11], who have reported that the greatest mortality during transport occurred in calves (0.296%), followed by dairy cows (0.207%) and feeder cattle (0.058%), with the lowest mortality in finished cattle (0.017%). It could be explained by younger animals having reduced capacity for thermoregulation, higher metabolic demands, an immature immune system and increased susceptibility to dehydration, fatigue, and handling stress during transport [7,10,13]. These physiological constraints may help to explain the elevated daily mortality observed in the Road treatment, which typically involve greater environmental challenges, such as snow in winter, and more frequent motion events (depending on the route, abrupt braking, vibration and acceleration) [2,22]. In addition, for biosecurity reasons (in accordance with border inspection, no hay or feed residues originating from the neighboring country are allowed inside the truck), the animals cannot be provided with feed or water during their transit through Argentina. Foot and Mouth Disease can be transmitted through contaminated feed: Argentina was recognized by some governments as being partially free from this in 2025 [23], whereas Chile has long been free of the disease. The Argentinian provinces free of FMD are Santa Cruz, Chubut and parts of Rio Negro and Neuquen, coinciding with the route taken by trucks through Argentina on the way to Osorno. However, feed supplies cannot easily be guaranteed as being from this region, leading to difficulties with providing disease-free feed for cattle in transit.

Among the factors associated with increased mortality in both juvenile and adult animals, the season in which the trip is performed influences the probability of death on a long-distance journey. With summer as the reference season, transport during winter was associated with an increase in the odds of mortality. However, the wide OR’s confidence intervals in all the categories do not allow us much confidence in the influence of the season on the probability of death. Further studies with greater statistical power and more journeys are needed to better characterize the influence of the season on the risk of death in this animal category. This could include studies of thermal conditions and the type of vehicle used to transport cattle by Road. Covering the vehicle improved head and ear temperature preservation during transport of sheep in cold conditions [24].

The distance traveled plays an important role in the mortality risk [11,13], with longer distances associated with higher mortality rates. Vecerek et al. [20] found that dairy cows transported long distances (up to 200 km) had substantially higher mortality rates (0.110%) compared with those on journeys not exceeding 50 km (0.019%). The present study substantially exceeded 200 km. Long-distance transport is therefore challenging for the animals [21]. However, in the present study, the cattle traveled similar distances by sea and land, but over a longer period for sea transport. Fluctuations in temperature, together with the lack of feed, water, and rest, are exacerbated by the duration of exposure and, consequently, by the length of the transport [21,25]. However, this may be a small effect compared with the other major differences between the two. In sea transport, the movement of the floor is steady, rhythmical and conceivably less stressful than Road transport, which may have sudden floor movement following braking, turning and acceleration. Thus, although sea journeys were characterized by longer durations, the relatively stable microclimatic conditions onboard vessels, together with a lower frequency of external disturbances such as sudden accelerations, braking events, and sharp turns, as well as limited access to feed and water, may contribute to a reduced daily mortality risk [17]. Accordingly, when mortality was standardized by journey duration, sea transport exhibited a lower relative risk compared with Road transport. Nevertheless, this metric should be interpreted with caution if overall mortality is the key consideration.

The climatic variation is likely to be less during the sea journey, compared with the passage through the Andes into, and out of, Argentina. The Andean region, through which the Road transport passes, experiences regular cold surges in winter, with temperatures at or below freezing and heavy snowfalls [26]. Ventilation of the cargo deck on the sea journey is minimal: just a series of thirty approximately 5 × 1 m ports on each side of the vessel, probably leading to the preservation of warmer ambient temperatures in winter than on the Road journey.

The results of this study reinforce the characterization of transport as a high-risk stage in the livestock supply chain, which is consistent with international evidence showing that mortality during transport may exceed on-farm mortality when expressed on a daily basis [3]. However, further research is needed to compare the corrected transport mortality rates reported here with on-farm daily mortality in Chile, given the apparent lack of reliable national data for routine farm-level mortality. An industry on-farm mortality study for calves in Chilean dairy farms estimated it to be 0.0035%/d [27], much less than the mortality rate of 0.14%/d recorded for calves in this study. Such comparisons are essential for contextualizing the relative contribution of transport to overall animal losses and for informing evidence-based policy, training, and regulatory improvements. The correction of mortality values according to journey duration provides a useful basis for comparing the relative risk associated with sea and road transport, particularly in situations involving different travel distances [2,22,23]. However, in this study, the mortality risk associated with two alternative transport routes from point A to point B was compared. The results indicate that road transport, due to its substantially shorter duration, is associated with fewer deaths than sea transport, despite exhibiting a higher mortality rate per day. This finding highlights the importance of journey duration as a key determinant of welfare outcomes, as prolonged transport is known to negatively affect the condition of transported animals [24,25].

The time-adjusted mortality patterns reinforce the characterization of road transport as a critical stage within the livestock production chain and underscore the need to strengthen animal welfare practices, particularly with respect to calf management. Transport-related mortality is widely recognized as a key welfare indicator, and evidence demonstrates that journey duration, travel distance, and seasonal conditions significantly influence mortality risk in both older cattle and calves [25,26,27]. These findings provide a scientific basis for updating national guidelines [28], improving transport standards, and prioritizing interventions aimed at reducing mortality and safeguarding animal welfare during long-distance livestock transport in southern Chile. Standards for both ship and road transport should specify assessment of fitness to load, provision of bedding, stocking densities (reduced for long journeys), feed and water provision [29] and maximum duration of transport, as a minimum. Current Chilean regulations [28,30] specify the responsible person for supervising loading and unloading; usually, the driver and this person must have a certificate of attendance at a training course. No bedding is mandated and a minimum stocking density of 500 kg/m^2^ is allowed.

In the present study, two alternative transport modalities connecting the same origin and approximately the same destination were compared. Under this comparative framework, the effective risk of mortality per journey was higher for sea transport than for road transport. Thus, despite the lower time-adjusted mortality observed during sea transport, the substantially longer journey duration resulted in a greater cumulative mortality risk per trip. These findings highlight that, if transport-related mortality between alternative routes serving the same origin and destination substantially exceeds those measured on the farm, the overall risk per journey may constitute a more appropriate and informative metric than mortality rates standardized solely by travel time.

## 6. Conclusions

Mortality was greater for calves than older cattle, especially when traveling by sea; however, when data was corrected for the journey duration, road transport had higher risks of mortality per day than ship transport. Winter was the season with the highest mortality, and high stocking densities increased the mortality of older cattle. Overall, the results demonstrate that types of transportation and management practices are critical determinants of transport-related mortality, highlighting the need for evidence-based improvements in current transport conditions to minimize losses and protect animal welfare.

## Figures and Tables

**Table 1:** Summary of truck floor area, animal load and route type for single and double deck trucks (one and two floors) used in the long-distance journeys by land and sea.

	Types of Truck												
	Truck (1 Floor)					Truck (2 Floor)							
Total floor area (m^2^)	28	30	31	33	34	54	55	56	58	59	60	73	83
Average number of animals per journey	33	48	31	42	41	37	74	79	72	79	42	86	81
Floor area per animal (m^2^)	0.61	0.63	1.00	0.79	0.83	1.46	0.74	0.71	0.80	0.75	1.43	0.85	1.02
Mean weight transported(kg/m^2^)	363.7	379.6	339.5	343.3	347.6	165.3	304.5	319.5	320.3	314.7	219.5	285.0	281.4
Number of journeys completed	1	1	16	20	173 (160/13)	6	21	33	7	18	14	28	57
Treatment	Sea	Sea	Sea	Sea	Land/Sea	Sea	Sea	Sea	Sea	Sea	Sea	Sea	Land

**Table 2:** Descriptive data: number of journeys classified by type of trip, animal category, season, and number of journeys with at least one dead animal.

Type of Trip	Category	Season	Number of Trips	Number of Journeys with at Least One Dead Animal
Sea	Calves	Summer	0	-
		Autumn	108	26
		Winter	27	11
		Spring	9	2
	Juveniles and adults	Summer	6	1
		Autumn	19	5
		Winter	6	3
		Spring	3	0
Land	Calves	Summer	10	1
		Autumn	62	7
		Winter	30	3
		Spring	13	0
	Juveniles and adults	Summer	30	0
		Autumn	41	1
		Winter	14	3
		Spring	17	2

**Table 3:** Logistic regression model * to assess the probability of death in long distance calves’ trips.

Variable	Mean ± SD	Odds Ratio	95% CI (Lower)	95% CI (Upper)	*p*-Value
Intercept	-	0.947	0.279	4.542	0.940
Mode of trip					
Sea	-	Reference			
Land	-	0.267	0.123	0.538	<0.001
Available area per animal (m^2^/animal)	0.80 ± 0.42	0.307	0.041	2.175	0.198

* Hosmer–Lemeshow test, *p*-value = 0.19. AUC value = 0.69.

**Table 4:** Logistic regression model * to assess the probability of death in long distance juvenile and older cattle trips.

Variable	Mean ± SD	Odds Ratio	95% CI (Lower)	95% CI (Upper)	*p*-Value
Intercept	.	0.000443	0.0000006184	0.04889	0.006
Mode of trip					
Sea	.	Reference			
Land	.	0.109	0.0255	0.3993	0.001
Season					
Summer	.	Reference			
Autumn	.	3.127	0.449	63.159	0.319
Winter	.	23.11	2.742	535.612	0.012
Spring	.	8.414	0.635	218.087	0.118
Weight transported per unit of space (kg/m^2^)	334.01 ± 58.85	1.016	1.0035	1.0333	0.032

* Hosmer–Lemeshow test, *p*-value = 0.38. AUC value = 0.87.

**Table 5:** Descriptive data of the journeys that resulted in the death of one or more animals, adjusted by duration according to the type of trip.

Trip	Animal Category	Number of Journeys with 1 or More Mortality	Number of Dead Animals	Total Deaths Adjusted by Number of Days	Adjusted Mean Deaths per Day
Sea	Calves	39	50	12.5	0.32
	Older cattle	9	9	2.25	0.25
	Total	48	59	14.7	0.31
Land	Calves	11	22	11.0	1.0
	Older cattle	6	8	4.0	0.6
	Total	17	30	15.0	0.88

## Data Availability

The data presented in this study are not publicly available due to confidentiality agreements with the commercial livestock transport company that provided access to the records. Data may be available from the corresponding author upon reasonable request and with permission from the company.

## Appendix A

**Table A1:** Monthly and seasonal averages of air temperature (°C) and relative humidity (%) in Los Lagos, Aysén, and Magallanes regions.

	Regions of Chile					
	Los Lagos		Aysén		Magallanes	
	T (°C)	RH (%)	T (°C)	RH (%)	T (°C)	RH (%)
January	16	76	14	59	12	60
February	17	76	14	59	12	62
March	14	79	11	66	9	65
Summer	15	77	13	61	11	63
April	12	83	8	71	7	69
May	9	85	4	77	5	75
June	8	85	1	80	3	77
Autumn	10	84	4	76	5	73
July	7	84	1	80	2	76
August	8	82	4	73	4	73
September	9	80	6	67	5	66
Winter	8	82	4	73	4	72
October	10	79	8	61	8	62
November	10	77	10	62	9	61
December	13	73	13	59	11	60
Spring	11	77	11	61	9	61

## References

[1] Broom D.M. (2003). Transport stress in cattle and sheep with details of physiological, ethological and other indicators. Dtsch. Tierarztl. Wochenschr..

[2] Buckham-Sporer K., Earley B., Marti S. (2023). Current knowledge on the transportation by road of cattle, including unweaned calves. Animals.

[3] Bachelard N. (2022). Animal transport as regulated in Europe: A work in progress as viewed by an NGO. Anim. Front..

[4] Sales F., Bravo-Lamas L., Realini C.E., Lira R., Aldai N., Morales R. (2020). Grain supplementation of calves as an alternative beef production system to pasture-finished steers in Chilean Patagonia: Meat quality and fatty acid composition. Transl. Anim. Sci..

[5] Arias R. (2024). Beef production and the beef evaluation system in Chile: Description, characterization, and quality. Anim. Front..

[6] Keane M.G., Allen P. (1998). Effects of production system intensity on performance, carcass composition and meat quality of beef cattle. Livest. Prod. Sci..

[7] Swanson J.C., Morrow-Tesch J. (2001). Cattle transport: Historical, research, and future perspectives. J. Anim. Sci..

[8] Earley B., Murray M., Prendiville D.J. (2010). Effect of road transport for up to 24 hours followed by twenty-four-hour recovery on live weight and physiological responses of bulls. BMC Vet. Res..

[9] Gupta S., Earley B., Crowe M.A. (2007). Effect of 12-hour road transportation on physiological, immunological and haematological parameters in bulls housed at different space allowances. Vet. J..

[10] Todd S.E., Mellor D.J., Stafford K.J., Gregory N.G., Bruce R.A., Ward R.N. (2000). Effects of food withdrawal and transport on 5- to 10-day-old calves. Res. Vet. Sci..

[11] Simova V., Voslarova E., Vecerek V., Passantino A., Bedanova I. (2017). Effects of travel distance and season of the year on transport-related mortality in cattle. Anim. Sci. J..

[12] González L.A., Schwartzkopf-Genswein K.S., Bryan M., Silasi R., Brown F. (2012). Relationships between transport conditions and welfare outcomes during commercial long-haul transport of cattle in North America. J. Anim. Sci..

[13] Malena M., Voslářová E., Tomanová P., Lepková R., Bedáñová I., Večerek V. (2006). Influence of travel distance and the season upon transport-induced mortality in fattened cattle. Acta Vet. Brno.

[14] Teke B. (2013). Shrink and mortality of beef cattle during long distance transportation. Anim. Welf..

[15] Bravo V.M., Knowles T.G., Gallo C. (2020). Transport, associated handling procedures and behaviour of calves marketed through chilean auction markets. Animals.

[16] Tadich N., Gallo C., Brito M.L., Broom D.M. (2009). Effects of weaning and 48 h transport by road and ferry on some blood indicators of welfare in lambs. Livest. Sci..

[17] Navarro G., Bravo V., Gallo C., Phillips C.J. (2019). Physiological and behavioural responses of cattle to high and low space, feed and water allowances during long distance transport in the south of Chile. Animals.

[18] Phillips C.J.C., Grandin T. (2024). Transport of cattle, sheep and other livestock by sea and air. Livestock Handling and Transport.

[19] Department of Agriculture, Fisheries and Food Reports to Parliament. https://www.agriculture.gov.au/biosecurity-trade/export/controlled-goods/live-animals/live-animal-export-statistics/reports-to-parliament.

[20] Department of Agriculture and Water Resources, Australian Government Review of the Australian Standards for the Export of Livestock: Sea Transport. Final Report. Technical Advisory Committee. https://www.agriculture.gov.au/sites/default/files/sitecollectiondocuments/animal/review-asel-sea-transport-final-report.pdf.

[21] Carnovale F., Arney D.R., Lowe D., Phillips C.J.C. (2025). Animal welfare incidents during and after transport to Australian export slaughterhouses: An evaluation of government reports (2020–2021). Vet. Rec. Open.

[22] Nicolaisen S., Langkabel N., Thoene-Reineke C., Wiegard M. (2023). Animal welfare during transport and slaughter of cattle: A systematic review of studies in the European legal framework. Animals.

[23] Government of Canada (2025). Foot and Mouth Disease—Countries Officially Recognized by Canada as Free of the Disease.

[24] Carnovale F., Xiao J., Shi B., Kaart T., Arney D., Phillips C.J.C. (2021). The effects of vehicle type, transport duration and pre-transport feeding on the welfare of sheep transported in low temperatures. Animals.

[25] Nielsen B.L., Dybkjaer L., Herskin M.S. (2011). Road transport of farm animals: Effects of journey duration on animal welfare. Animal.

[26] Garreaud R.D. (2009). The Andes climate and weather. Adv. Geosci..

[27] Sanhueza J.M., Lembeye F. (2021). Mortality Rate in Dairy Calves in Chile and the Association with Waste Milk Feeding, a Pilot Study. Engormix. https://en.engormix.com/dairy-cattle/calf-health/mortality-rate-dairy-calves_a47118/.

[28] Ministerio de Agricultura (2013). Aprueba Reglamento Sobre Protección del Ganado Durante el Transporte.

[29] EFSA Panel on Animal Health and Welfare (AHAW) (2022). Welfare of cattle during transport. EFSA J..

[30] Subsecretaría de Salud Pública (2009). Ley Chile-Ley 20380-Biblioteca del Congreso Nacional de Chile.

